# *Bartonella* and *Rickettsia* Infections in Haematophagous *Spinturnix myoti* Mites (Acari: Mesostigmata) and their Bat Host, *Myotis myotis* (Yangochiroptera: Vespertilionidae), from Poland

**DOI:** 10.1007/s00248-018-1246-5

**Published:** 2018-08-27

**Authors:** Agnieszka Szubert-Kruszyńska, Joanna Stańczak, Stella Cieniuch, Edyta Podsiadły, Tomasz Postawa, Jerzy Michalik

**Affiliations:** 1 Evolutionary Biology Group, Faculty of Biology, Adam Mickiewicz University, Poznan, Poland; 2 Department of Tropical Parasitology, Institute of Maritime and Tropical Medicine, Medical University of Gdańsk, Gdynia, Poland; 3 Department of Laboratory Diagnostics and Clinical Immunology of Developmental Age, Public Pediatric Teaching Hospital, Warszawa, Poland; 4 Institute of Systematic and Evolution of Animals, Polish Academy of Sciences, Krakow, Poland; 5 Department of Animal Morphology, Faculty of Biology, Adam Mickiewicz University, Umultowska 89, 61-614 Poznan, Poland

**Keywords:** *Bartonella*, *Rickettsia*, *Anaplasma phagocytophilum*, Bats, Spinturnicidae

## Abstract

Hematophagous *Spinturnix myoti* mites and their host, the greater mouse-eared bat (*Myotis myotis*), were tested for the presence of *Bartonella* spp., *Rickettsia* spp., and *Anaplasma phagocytophilum*. In total, *Bartonella* spp. DNA was amplified in 28% of 134 mite pools and in 25% of 59 bats tested by PCR targeting a fragment of citrate synthase *gltA* gen. Adult mites were at least threefold more frequently infected compared to immature stages. The overall infection prevalence among mite pools from cave-dwelling bats was higher than for those collected from attic shelters. Three distinct genotypes were detected. The most prevalent genotype in mites and bats matched closely with *Candidatus* Bartonella hemsundetiensis identified in bats from Finland and was relatively distant from bat-borne *Bartonella* strains described in the UK and France. Importantly, most sequences were close to those reported in forest workers from Poland. The presence of identical genotype among *S. myoti* samples and *M. myotis* bats suggests that bartonellae can be shared between mites and their bat hosts. In this case, wing mites could serve as vectors, whereas their hosts as reservoirs. One blood sample was positive by PCR for the *msp2* gene of *A. phagocytophilum*. Two mite pools yielded *Rickettsia* spp. DNA. Both sequences were distinct from any known species but can be classified as spotted fever group *Rickettsia* spp. Our findings expanded our knowledge on the role of spinturnicid mites in the ecology and epidemiology of bacterial infections associated with vespertilionid bats, especially regarding the genus *Bartonella*.

## Introduction

In the ecology and epidemiology of vector-borne zoonotic diseases, it is essential to identify (i) a group of vertebrate reservoir species acting as a natural source for a pathogen and (ii) competent biological vectors which ensure its multiplication, long-term maintenance, and finally active transmission to hosts. These components are responsible for emergence and maintenance of endemic foci of vector-borne diseases. Bats from the order Chiroptera are highly mobile flying vertebrates which comprise one fourth of the world’s species of mammals and frequently host heavy loads of highly specialized groups of hematophagous arthropods. Bat-adapted flies (Nycteribiidae, Streblidae), fleas (Ischnopsyllidae), bugs (Cimicidae), mites (e.g., Macronyssidae, Spinturnicidae), and ticks (Ixodidae and Argasidae) may carry various zoonotic pathogens and probably act as their vectors within chiropteran populations [1].

Mites of the family Spinturnicidae (Acari, Mesostigmata) belong to the most abundantly and regularly recorded non-tick acarines associated with bats from the suborder Yangochiroptera and with two families (Rhinopomatidae, Rhinolophidae) from the suborder Yinpterochiroptera, As permanent ectoparasites (without free-living stages) exhibit high host specificity completing their entire life cycle on one or a few closely related bat species [2, 3], they infest only bare fragments of bat’s skin like wing and tail membranes and therefore are called wing mites. Their reproduction period is strictly synchronized with the breeding cycle of the host. Consequently, the highest numbers of mites with prevalence values up to 100% are recorded on pregnant and lactating females, and especially on their offspring, which in Europe are nursed for several summer weeks (from June to July) in colonial roosts [3–5]. Whereas eggs and larval stages develop inside a pregnant female mite, each of active mobile developmental stages (the protonymph, deutonymph, and adult) feeds several times on the host’s blood and lymph [6]. To date, in Europe, the family Spinturnicidae comprises 15 species [7] and 13 of them have been confirmed on bats in Poland [8].

Repeated feedings and frequent co-infestation of European bats by bat-associated tick species, such as *Argas vespertilionis*, *Ixodes vespertilionis*, *I. ariadnae*, and occasionally *I. ricinus*, strongly imply that these mites are naturally exposed to various blood-borne bacterial agents [9–11]. However, the potential role of Spinturnicidae as biological vectors, reservoirs, or amplifiers for bacterial agents circulating in bat communities remains still poorly studied. There are only two published reports on bacterial agents detected in wing mites. Revees et al. [12] confirmed the presence of the 16S rRNA and p44 genes identical to *Anaplasma phagocytophilum* in *Spinturnix psi* collected from a bat of the genus *Miniopterus* in Madagascar. The sequence of the p44 gene showed 100% identity to those found in human and horse isolates with granulocytic anaplasmosis. Furthermore, Hornok et al. [9] identified *Bartonella* spp. DNA in *Spinturnix myoti* pools obtained from three *Myotis myotis* bats in Hungary.

The aim of our work was to investigate the presence of potentially zoonotic agents such as *Bartonella* spp., *Rickettsia* spp., and *Anaplasma phagocytophilum* in *Spinturnix myoti* mites and their main host, the greater mouse-eared bat, *Myotis myotis*, by PCR assays. These intracellular bacteria are predominantly transmitted by hematophagous arthropod vectors including mites and ticks [1]. The mode of transmission of these agents among populations of European bat species as well as vector competency of their highly specialized ectoparasites remains unknown. The greater mouse-eared bat is one of the largest European bats from the insectivorous family Vespertilionidae. During its reproduction period, it resides in caves or in large buildings, including church or school attics where it forms breeding aggregations, and therefore besides *Eptesicus serotinus*, it is recognized as the most common synanthropic species [13, 14]. It is parasitized with the *Spinturnix myoti* mite which may also occur on a few other *Myotis* species and only sporadically on bats of genera *Barbastella*, *Pipistrellus*, *Plecotus*, and *Vespertilio* [14]. This wing mite has a wide distribution range in Europe and additionally has been recorded in North Africa and Asia [15]. In the present study, bats were sampled for peripheral blood and mites in cave and attic roosting shelters localized in west-central and southern Poland.

## Materials and Methods

### Study Sites

**Fig. 1:**
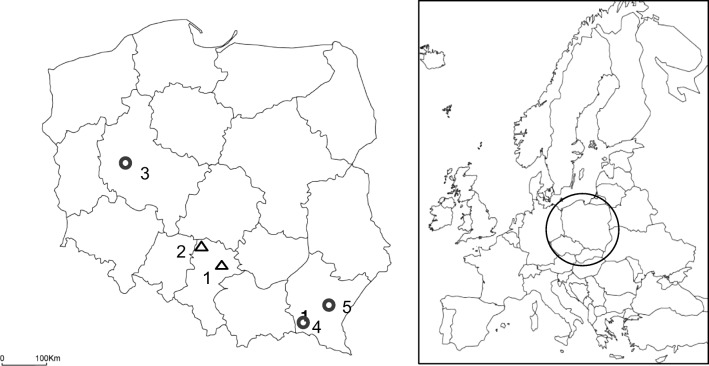
Location of the five collection sites where bats were sampled in Poland: (1) Studnisko cave, (2) Szachownica cave, (3) Kopanki (school), (4) Skalnik (church), and (5) Nowosielce (church). Triangles denote caves, whereas circles show attic shelters

Bats were sampled in five shelters of *Myotis myotis* located in west-central and southern Poland in years 2007–2008 (Fig. 1). The chosen shelters were represented by two caves: (1) Studnisko cave (Krakowsko-Wieluńska Upland, 346 m a.s.l., 19° 16′ E; 50° 43′ N) and (2) Szachownica cave (Śląsko-Krakowska Upland, 215 m a.s.l., 18° 48′ E; 51° 03′ N), and three attics: (3) a school attic in Kopanki (Wielkopolska Province, west-central Poland, 108 m a.s.l., 16° 18′ E, 52° 17′ N) and two church attics situated in (4) Skalnik and (5) Nowosielce (the Beskid Mountains, a part of the Carpathians: 350 m a.s.l., 21° 28′ E, 49° 34′ N, and 330 m a.s.l., 22° 23′ E, 50° 03′ N, respectively).

### Bat Capture and Mite Collection

In this study, only bats infested with spinturnicid mites were analyzed. Most of them (72 out of 80) were captured during their breeding season (from mid-May to mid-August) by hand directly from aggregations in attics or using mist nets and harp traps at colony entrances of caves. Eight individuals from the Szachownica cave were mist-netted during their mating period in October. Bats were placed into clean separate bags and identified based on differences in their morphological characteristics [16]. Sex and age (adult, juvenile) were determined for each caught individual. EDTA-blood samples (approximately 5 μL) were successfully collected from 59 of 80 (73.7%) bats by a puncture of the peripheral uropatagium vein using a 28-gauge needle and a pipette (Table 2). A fast acting gel (Super Clot Gel, Synergy Labs) was applied to the puncture site to disinfect it and avoid bleeding. Blood samples were stored at − 20 °C in Eppendorf tubes until molecular surveys. Spinturnicid mites were collected from wing and tail membranes of their hosts using forceps and preserved in 75% ethanol for further analyses. Their morphological identification was based on the keys by Dusbábek [17].

After collection of mites and blood, each animal was released in its natural habitat. Trapping and handling procedures of bats were approved by the permission of the Polish Ministry of Environment (no. DLOPiK-op/Ozgi-4200/IV.D-20/7666/06/aj). Due to the low number of adult males (*n* = 4), only adult females and juvenile bats were analyzed for differences in mean intensity calculated as mean number of mites per infested host ± standard deviation (SD) during the breeding season. The eight animals from the Szachownica cave, which were mist-netted in the postbreeding period in October, were excluded from the statistical analysis regarding infestation parameters.

### DNA Extraction, PCR Assays, and Sequencing

DNA was extracted from blood samples of bats and from spinturnicid mites by use of the commercial kits: Genomic Mini AX Blood and Sherlock AX (A&A Biotechnology, Gdynia, Poland), respectively. Mites were processed in pools containing between two and ten individuals derived from the same bat. Two groups of mites: (i) immature stages (protonymphs and deutonymphs) and (ii) adult stages were polled and tested separately. A total of 130 DNA samples was obtained from spinturnicid mites.

Samples were analyzed for the presence of *Bartonella* spp., *Rickettsia* spp., and *A. phagocytophilum* by conventional PCR assays. *Bartonella* spp. were detected using primers with primers BhCS781 and BhCS1137 targeting the citrate synthase *gltA* gen fragment with expected size 379 bp and under the cycling conditions as previously described [18]. Rickettsial DNA was detected using the RpCs.877 and RpCs.1258 primers amplifying a 381-bp fragment of the *gltA* gene which has conserved regions shared by all known *Rickettsia* species [19]. A species-specific direct PCR assay that amplifies a 334-bp fragment of the *msp2* gene (primers: MSP2-3f and MSP2-3r) was used for the detection of *A. phagocytophilum* [20, 21]. To determine genetic variants of *A. phagocytophilum*, a nested PCR amplifying a 546-bp region of the 16S rRNA gene was used as previously reported [22]. Positive and negative (doubled distilled water) controls were included with each PCR. PCR products were separated by 1.5% agarose gel electrophoresis and visualized by ethidium bromide. Selected PCR-positive products were sequenced in both directions using PCR primers. Sequencing products were resolved by using an ABI 3100 automated sequencer (PerkinElmer). Sequence analysis was performed by using the software package ABI Prism DNA Sequencing Analysis Software version 3.0 (PerkinElmer). All obtained sequences were compared with those available in GenBank databases using BLAST (www.ncbi.nlm.nih.gov/blast/Blast.cgi). Phylogenetic dendrograms with *Bartonella* spp. and *Rickettsia* spp. *gltA* sequences obtained in this study and selected sequences deposited in GenBank were constructed by the neighbor-joining algorithm method using CLC Sequence Viewer version 7.6. Jukes-Cantor model was used for nucleotide distance measurement. Representative partial sequences determined in this study were deposited in GenBank under the following accession numbers: JQ695834-40 for the *gltA* gene of *Bartonella* spp. and JQ695832 and JQ695833 for the *gltA* gene of *Rickettsia* spp. Rates of infection were analyzed by use of chi-squared test *χ*^2^, whereas differences in mean intensity of mite infestation by Mann-Whitney *U* test with *P* < 0.05 were considered statistically significant.

## Results

Altogether, 80 individuals of the greater mouse-eared bat were included in this study: 43 adult females, 19 young females, four adult males, and 14 young males. A total of 707 spinturnicid mites, including 400 adults (56.6%) and 307 immature individuals (43.4%), were removed from the captured hosts (Table 1). All were identified as *Spinturnix myoti* Kolenati 1856.

**Table 1:** Prevalence of *Bartonella* spp. and *Rickettsia* spp. in *Spinturnix myoti* mites depending on the collection sites and developmental stages (mites were tested in pools)

Shelter (No. bats infested)	Mite stage	No. mites	No. pools tested	*Bartonella* spp.	*Rickettsia* spp.
				No. (%) positive pools	No. (%) positive pools
1. Studnisko cave (42)	Adult	234	42	22 (52.4)	2 (4.8)
	Immature	223	40	6 (15.0)	0
	Subtotal	457	82	28 (34.1)	2 (2.4)
2. Szachownica cave (8)	Adult	20	7	3 (42.9)	0
	Immature	4	1	0	0
	Subtotal	24	8	3 (37.5)	0
3. Kopanki school attic (20)	Adult	75	18	6 (33.3)	0
	Immature	55	13	1 (7.7)	0
	Subtotal	130	31	7 (22.6)	0
4. Skalnik church attic (5)	Adult	40	5	0	0
	Immature	18	2	0	0
	Subtotal	58	7	0	0
5. Nowosielce church attic (5)	Adult	31	5	0	0
	Immature	7	1	0	0
	Subtotal	38	6	0	0
Total (80)	Adult	400	77	31 (40.3)	2 (2.6)
	Immature	307	57	7 (12.3)	0
		707	134	38 (28.4)	2 (1.5)

### Intensity of Mite Infestation

Mean (± SD, range) mite intensities assessed for adult females (*n* = 43) and juvenile bats (including 15 females and 11 males) during the breeding season were similar (9.8 ± 4.6 and 8.9 ± 4.0, respectively). Cave-dwelling bats hosted higher mite loads than those from attics (10.9 ± 3.9 vs. 7.5 ± 4.0 per bat, respectively, Mann–Whitney *U* test *p* = 0.0004).

### Bacterial Infections in Mites

DNA of *Bartonella* spp. was amplified in 38 (28.4%) of the 134 *S. myoti* pools (Table 1). The positive samples were collected from 35 (43.7%) of the 80 bats, including 23 (48.9%) out of 47 adults and 12 (36.4%) out of 33 juveniles. Adult mites were at least threefold more frequently infected with the bacterium compared to immature stages (40.3 and 12.3%, respectively, *χ*^2^ test *P* = 0.0003). The overall infection prevalence among mite pools removed from cave-dwelling bats was significantly higher than in those collected from bats in attic shelters (34.4 and 15.9%, respectively; *χ*^2^ test *P* = 0.025). Animals infested with at least one PCR positive pool were also more frequent in the former group in comparison with bats from the latter (56.0 vs. 23.3%; *χ*^2^ test *P* = 0.004). Three animals from Studnisko cave were concurrently infested with infected adult and immature stages of *S. myoti*.

**Fig. 2:**
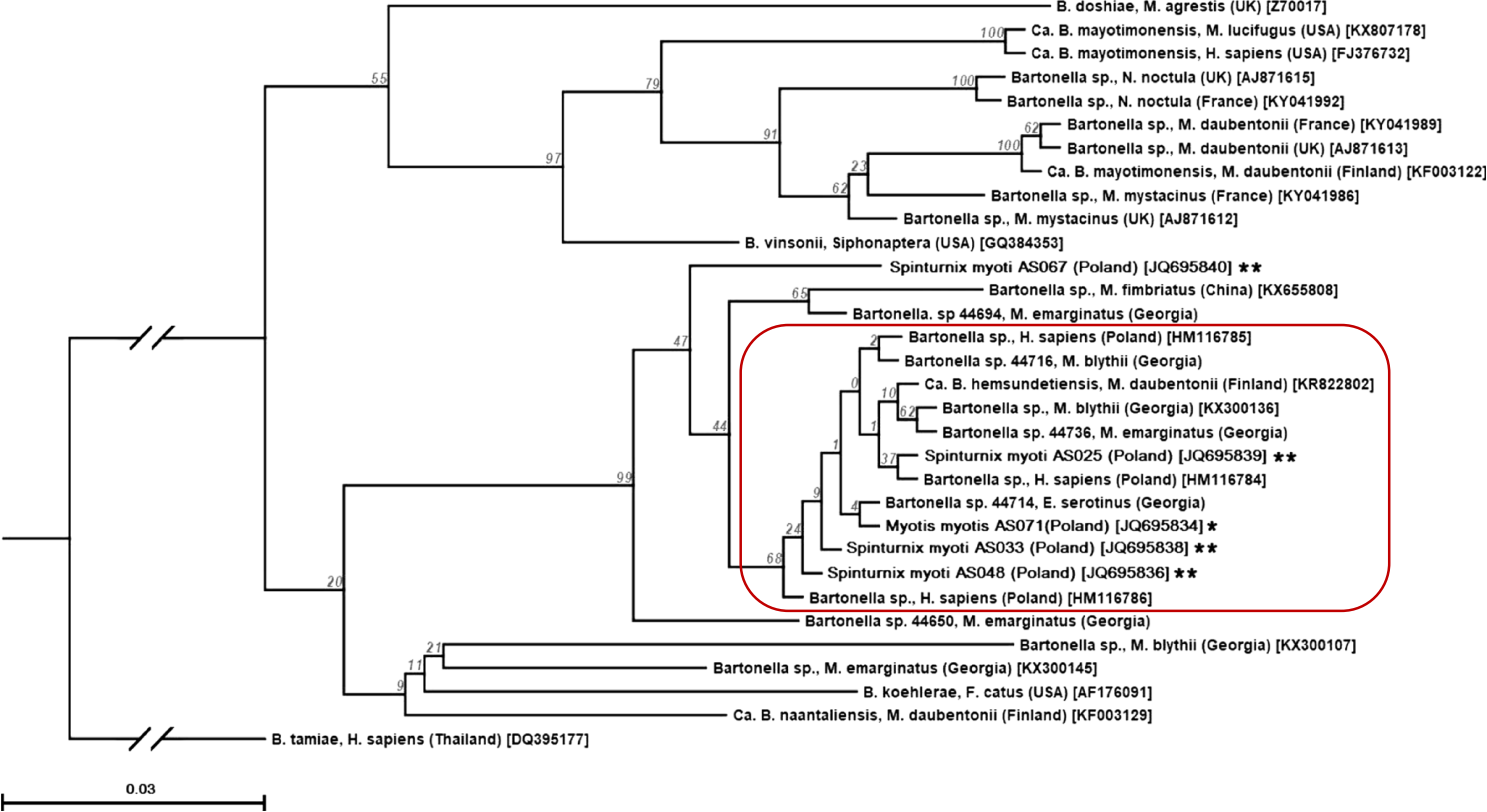
Phylogenetic relationships of *Bartonella* spp. genotypes found in *Spinturnix myoti* mites and *Myotis myotis* bats based on the fragment of *gltA* gene of *Bartonella* spp. and selected sequences available from GenBank. Inference was made by using the neighbor joining method in CLC Sequence Viewer Version 7.6. Jukes-Cantor model was used for nucleotide distance measurement. Bootstrap analysis with 100 replicates was performed. The scale represents 0.03 substitution per base per indicated horizontal distance. Note: branches shorter than 0.0022 are shown as having length 0.0022. The gapped branches have been truncated to one third of their original length for clarity. The tree is rooted with *gltA* gene of *B. tamiae* [DQ395177]. *Bartonella* spp. genotypes found in blood of bats and in mite pools in this study were marked with one and two asterisks, respectively

Three novel *gltA* gene sequences of *Bartonella* spp. termed as genotypes A, B, and C were successfully generated from representative PCR positive mite pools (Table 3). Their nucleotide identity to each other ranged from 96.3 to 99.7%. The genotype A proved to be the most common and was identified in 18 (90%) out of 20 PCR amplicons, whereas the variants B and C were detected only in two single pools. The dominant genotype A shared 100% (299/299 bp) identity with *Bartonella* strains isolated from three bat species: *Myotis blythii* (GenBank e.g., KX300136), *M. emarginatus* (isolate B44736), and *Eptesicus serotinus* (isolate B44714) sampled in Georgia. Furthermore, the genotypes A and B exhibited 100 and 99.4% similarity to human-derived sequences identified in forest workers in Poland (e.g., GenBank HM116786). Both variants were also closely related to the recently reported *Candidatus* Bartonella hemsundetiensis (GenBank KR822802) from *M. daubentonii* bats captured in Finland (mean sequence similarity = 99.5%). The variant C reached 96.3% nucleotide identity. Our samples were distinct from *Bartonella* spp. described from bats in the UK and France. Figure 2 shows the phylogenetic relationships of the *Bartonella* spp. *gltA* genetic variants detected in mites and bats in this study with selected sequences available on GenBank.

**Fig. 3:**
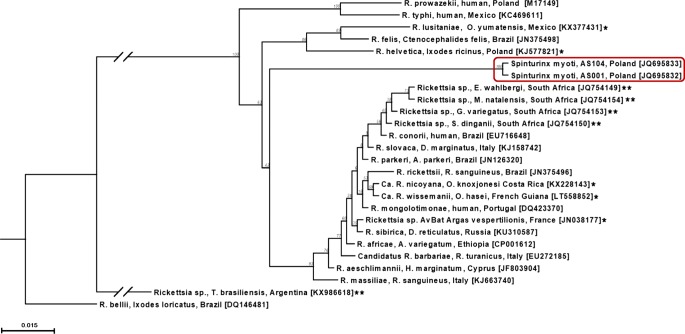
Phylogenetic relationships of the two *Rickettsia* genotypes found in *Spinturnix myoti* mites species based on the fragment of *gltA* gene of *Rickettsia* spp. and selected sequences available from GenBank. Inference was made by using the neighbor joining method in CLC Sequence Viewer Version 7.6. Bootstrap analysis with 100 replicates was performed. The scale represents 0.015 substitution per base per indicated horizontal distance. Note: branches shorter than 0.0015 are shown as having length 0.0015. The gapped branches have been shortened by 0.03 for clarity. The tree is rooted with *gltA* gene of *R. bellii* [DQ146481]. Selected *Rickettsia* spp. sequences from bat-associated ticks and several bat species were marked with one and two asterisks, respectively

*Rickettsia* spp. DNA was detected in two (1.5%) pools comprising adult mites (Table 1). Sequence analysis of both positive products with *gltA*-specific primers revealed that they were identical to one another and unique from other known *Rickettsia* spp., with DNA similarity values < 92% (GenBank acc. nos. JQ695832, JQ695833). They showed maximum similarity score of 91.8% (312/340 bp) with *R. massiliae* (KJ663740) and *Candidatus* R. barbariae (EU272185) detected in *Rhipicephalus sanguineus* and *R. turanicus* ticks, respectively. Moreover, both samples shared 91.5% similarity with the *Rickettsia* sp. AvBat strain isolated from *Argas vespertilionis* in France (JN038177), and 91.2% similarity with *Candidatus* Rickettsia wissemanii identified in *Ornithodoros hasei* in French Guiana (LT558852) and *Candidatus* Rickettsia nicoyana isolated from *Ornithodoros knoxjonesi* in Costa Rica (KX228143). Bats are hosts for the mentioned ticks. The *gltA* phylogenetic tree reflected these relationships with a distinct position of both our sequences which clustered in a separate clade (Fig. 3). All mite samples tested negative for DNA of *A. phagocytophilum* by using primers targeting the *msp2* gene.

### Bacterial Infections in Bats

*Bartonella* spp. DNA was detected in 15 (25.4%) of the 59 blood samples, with the range from 12.5 to 29.7% depending on the collection site (Table 2). Blood PCR positive animals were recorded both in cave and in school attic shelters. The infection prevalence was comparable among juvenile and adult bats (22.2 and 28.1%, respectively). Of the 15 bacteremic animals, 11 (73%) carried also PCR-positive mite pools. Moreover, two of the 11 bats hosted concurrently infected adult and immature *S. myoti* stages. Sequence analysis of 10 selected *gltA* gene sequences identified in the bats demonstrated 100% identity to the genotype A found in most infected mite pools (Table 3). Two representatives of the *gltA* sequences from *M. myotis* were submitted to the GenBank (JQ695834, JQ695835).

**Table 2:** Prevalence of *Bartonella* spp. in *Myotis myotis* bats depending on the collection sites and age/sex groups

Shelter	Adults			Juveniles		Total (ad + juv)	
		No. tested/positive (%)		No. tested/positive (%)		No. tested/positive (%)	
Studnisko cave	Female	22	6 (27.3)	6	2 (33.3)	28	8 (28.6)
	Male	3	2 (66.7)	6	1 (16.7)	9	3 (33.3)
	f + m	25	8 (32.0)	12	3 (25.0)	37	11 (29.7)
Szachownica cave	Female	0	0	4	0	4	0
	Male	1	0	3	1 (33.3)	4	1 (25.0)
	f + m	1	0	7	1 (14.3)	8	1 (12.5)
Kopanki school attic	Female	6	1 (16.7)	6	2 (33.3)	12	3 (25.0)
	Male	0	0	2	0	2	0
	f + m	6	1 (16.7)	8	2 (25.0)	14	3 (21.4)
Total	Female	28	7 (25.0)	16	4 (25.0)	44	11 (25.0)
	Male	4	2 (50.0)	11	2 (18.2)	15	4 (26.7)
	f + m	32	9 (28.1)	27	6 (25.2)	59	15 (25.4)

**Table 3:** Prevalence of three *gltA* gene genotypes of *Bartonella* spp. detected in *Spinturnix myoti* mites and in blood of their hosts, *Myotis myotis* bats

Species	Locality	Distribution of genotypes A,B,C among mite and bat samples (age/sex)*		
		A	B	C
*S. myoti*	Studnisko cave	AS007(5ad), AS008(4juv)	**AS025**(6ad)	
		AS012(5juv), AS017(5ad)		
		**AS033**(5ad), **AS036**(6ad)		
		AS038(6ad), AS084(5ad)		
		AS085(5juv), AS088(5ad)		
		AS090(6ad), AS094(8ad)		
	Szachownica cave	AS068(1ad), AS069(3ad)		**AS067**(3ad)
	Kopanki attic	AS043(5juv), **AS048**(4ad)		
		AS052(3ad), AS059(3ad)		
*M. myotis*	Studnisko cave	AS024(f.juv), AS029(f.juv)		
		AS038(f.ad), AS040(f.ad)		
		AS065(f.ad), **AS071**(f.ad)		
		AS073(f.ad), AS074(m.ad)		
	Szachownica cave	AS063(m.juv)		
	Kopanki attic	**AS050**(f.juv)		

*Representative *gltA* gene sequences of *Bartonella* spp. submitted to GenBank in boldface; mites were studied in pools. AS033: JQ695838, AS036: JQ695837, AS048: JQ695836, AS025: JQ695839, AS067: JQ695840, AS050: JQ695835, AS071: JQ695834

*Rickettsia* spp. DNA was not detected in any of the 59 blood samples. One female, derived from Kopanki school attic, yielded a 334-bp fragment of the *A. phagocytophilum* msp2 gene. Unfortunately, due to a limited volume of DNA extract from this single sample, a confirmation of the amplified *A. phagocytophilum* DNA by the 16S rRNA gene nested PCR assay and subsequent sequencing was unsuccessful.

## Discussion

In this report, we screened using PCR assays hematophagous *Spinturnix myoti* mites and their natural host, the greater mouse-eared bat, *Myotis myotis*, for the presence of medically important arthropod-borne intracellular bacteria: *Bartonella* spp., *Rickettsia* spp., and *A. phagocytophilum*. Among these agents, bartonellae proved to be the most common infection both in mites and their hosts. Overall, 28% *S. myoti* pools and 25% *M. myotis* blood samples yielded these bacteria. The presence of bartonellae in *S. myoti* mites was previously reported in a survey conducted in Hungary, in which each of the three pools tested (comprising 85 mites) by TaqMan PCR yielded *Bartonella* spp. [9].

The infection prevalence in mites sampled in our study varied depending on a developmental stage and a shelter type of their hosts. Pools with adult mites harbored the bacterium at least threefold more frequently than those with immature stages (40 vs. 12%). Given that spinturnicid mites feed multiple on an individual host for at least several months, these differences could be attributed to previous episodes of the adult mites which would have fed upon bacteriemic animals. In our opinion, detection of *Bartonella* DNA both in adult and subadult *S. myoti* pools may imply that spinturnicid mites are not only exposed to the infection but they might also maintain it in subsequent developmental stages (transstadial transmission). The presence of infected pools with adult mites collected from 18 blood-negative bats seems to support this supposition, especially that none of 13 immature mite pools, obtained from the same hosts, yielded the bacterium (data not shown). *Bartonella* species invade erythrocytes and endothelial cells causing usually long-lasting infections accompanied by a relapsing intraerythrocytic bacteremia of variable level which in their mammalian reservoir hosts can persist months or even years [23]. Therefore, spinturnicid mites, as permanent ectoparasites with multiple feeding mode observed in each life stage, have ample opportunities to encounter and acquire blood-borne pathogens during a bacteremic period of the host. Furthermore, their typically high abundance and prevalence up to 100% in summer maternity colonies [24], vertical (from mother to offspring) and horizontal (among individuals) transfer between tightly roosting animals, clearly increase their dispersal capacity and make them at least theoretically ideal vectors for various blood-borne bacterial agents. Of note is that the mean mite intensity values recorded on adult females and juvenile bats during the breeding season were comparable (10.6 and 8.9 mites per bat, respectively). This finding confirms previous results (e.g., [3, 4]) and demonstrates a high dispersion potential of wing mites which may facilitate transfer of blood-borne bacteria among bats.

Interestingly, mite samples from cave dwelling bats were twice more frequently infected than those collected from attic-dwelling hosts (34 vs. 16%). Similarly, in a study conducted by Sándor et al. [25], the highest prevalence of *Bartonella* spp. infected bat flies (Nycteribiidae) was recorded among bat species (*Rhinolophus euryale*, *Myotis capaccinii*, *Miniopterus schreibersii*) roosting exclusively in caves. Moreover, in our study, a higher proportion of cave-dwelling animals hosted PCR positive pools in comparison with those sampled in attics (56 vs. 27%). This indicates that bats roosting in caves colonies were more often exposed to infected mites that could be attributed to higher *S. myoti* loads recorded on animals breeding in this type of shelter. Although *M. myotis* can breed successfully in micro-climatically distinct nursery roosts [26], conditions prevailing in caves with year-round stable microclimate seem to be particularly favorable for a larger reproduction of spinturnicid mites and may explain differences in infestation parameters observed in our study. Postawa et al. [24] demonstrated that greater mouse-eared bats from cave nursery colonies harbored at least threefold more *S. myoti* mites than those from attic colonies, irrespective of host sex or age. The authors concluded that the microclimate of the host’s roosts favor ectoparasite abundance. In our opinion, higher loads of spinturnicid mites especially on bacteremic animals may not only increase the effectiveness of their horizontal and vertical transfer but might also contribute to higher prevalence of *Bartonella* spp. in *S. myoti* mites collected from cave-dwelling bats as we observed in the present study. Moreover, they may act as amplifiers for horizontal transmission of bat-associated *Bartonella* spp. to competent vectors (e.g., bat bugs and argasid ticks) which are known to attack occasionally humans [1, 9].

The detection of *Bartonella* spp. DNA in peripheral blood of 25% greater mouse-eared bats sampled in our study demonstrates their susceptibility to bartonellae infections which seem to be rather frequent in populations of this bat species. Comparison of available data regarding *Bartonella* infection among European bats from the family Vespertilionidae showed that the mean prevalence found in *M. myotis* was higher than the 8.8% reported in bats from the UK or the 10% described in France, but lower than that of 37% in Finland, and 38.7% in Georgia, a country in the Caucasus region located at the border of Eastern Europe [27–30]. These bat-borne *Bartonella* spp. have been found so far in ten vespertilionid bat species. Five of them belong to the genus *Myotis*: *M. blythii*, *M. daubentonii*, *M. emarginatus*, *M. mystacinus*, and *M. myotis* (this study). The remaining five species comprise: *Eptesicus nilssoni*, *E. serotinus*, *Pipistrellus nathusii*, *P. pygmaeus*, and *Nyctalus noctula*. Interestingly, in our study, the majority of blood-positive bats (73%) hosted concurrently infected mite pools, which implies that these mites might have acquired the bacterium during feeding upon bacteremic hosts. This indicates that *M. myotis* may develop active bacteremia and maintain the circulation of bartonellae acting as a competent reservoir. In our opinion, co-occurrence of infected adult and immature stages of *S. myoti* on two PCR-positive bats, additionally, supports this presumption. On the other hand, the finding that juvenile bats and adult females (excluding animals from Szachownica) showed identical infection rates (25%) suggests a potential role of spinturnicid mites in active acquisition and transmission of *Bartonella* spp. between mothers and their offspring during the reproductive season. This hypothesis appears to be strongly supported by the fact that PCR-positive *M. myotis* bats and infected mite pools yielded the same genotype A of the bacterium.

Analysis of the partial *gltA* gene sequences of *Bartonella* spp. found in *S. myoti* pools revealed the presence of three genotypes A, B, and C, with evident predominance of the former. Remarkably, the genotype A proved to be the only variant identified among bacteriemic *M. myotis* bats. The genotype A exhibited 100% similarity to *Bartonella* strains recently isolated from three bat species sampled in Georgia representing the largest genogroup Vesp-6 dominated by *M. blythii* [30]. Unexpectedly, all of the *Bartonella* genotypes identified in our study were phylogenetically distant from those found in bats from UK and France [27, 29]. They were also distant from strains reported from Finland [31] including *B. naantaliensis* and two strains detected in *M. daubentonii* and *E. nilssoni* closely resembling *Candidatus B. mayotimonensis* isolated from an endocarditis human patient in the USA [32]. On the other hand, the genotypes A and B in our study, as well as Georgian strains from Vesp-6 genogroup, clustered along with *Candidatus B. hemsundetiensis* recently detected by Lilley et al. [28] in blood of *M. daubentonii* bats from Finland. In our opinion, postglacial recolonization of Europe by several *Myotis* spp. from the region of Caucasus [33] might contribute to the observed phylogenetic similarity of *Bartonella* sequences among samples from *M. myotis* representing populations of Central Europe and Georgian bats from Ves-6 genogroup belonging to Caucasian populations.

It is noteworthy that the genotypes A and B were genetically similar to sequences previously detected in forest workers in Poland, however with unknown pathogenicity [34]. Although some bat-associated bartonellae described in the UK, Finland, and France resemble *Candidatus B. mayotimonensis*, an etiological agent of human endocarditis [32], pathogenic potential of these European strains, and significance in public or veterinary health remain still to be elucidated. To date, the *gltA*, *rpoB*, and ISR sequences identical with the endocarditis patient strain DNA have been identified only in the little brown myotis (*Myotis lucifugus*) sampled from Michigan. It seems to be possible that *M. lucifugus* is the only species acting as a natural reservoir for the human pathogen in North America [35]. Furthermore, *B. tamiae*, a newly described species isolated from patients in Thailand, has been detected in bat-associated *Ixodes vespertilionis*, Nycteribiidae flies, and bat spleens in Algeria [36]. However, the mode of potential transmission of bartonellae between bats and humans is still unclear.

We provide the first evidence of *Rickettsia* spp. in two pools of spinturnicid mites. Both identical sequences formed a unique clade comparing to other known species but can be classified as spotted fever group *Rickettsia* spp. Recently, *Rickettsia* species have been identified in several argasid tick species parasitizing bats in France, French Guiana, Mexico, Costa Rica, and the UK [11, 37–40]. However, Revees et al. [12] failed to amplify DNA of *Rickettsia* spp. in any of 17 pools comprising macronyssid and spinturnicid mites collected from bats mostly in North and Central America and Africa. In a Hungarian report, none of 436 mites representing the same families yielded the agent [9]. Therefore, we presume that *Rickettsia* spp. infections among bat-adapted mites and their hosts seem to be rather rare. The molecular presence of *Rickettsia* spp. in bats has been so far described only in two reports from South Africa and Brazil [41, 42]. Only one (1.7%) blood sample was positive by PCR for the msp2 gene of *A. phagocytophilum*, but the product could not be sequenced. On the other hand, the species-specific PCR with highly sensitive primer set allow us to claim that the positive sample could yield DNA of *A. phagocytophilum*. As far as we know, this is the first information indicating the possible presence of the bacterium in bats.

In conclusion, the occurrence of identical genetic variant of *Bartonella* among infected *S. myoti* samples and *M. myotis* bats strongly suggests that bartonellae can be shared between spinturnicid mites and their bat hosts. In this case, mites could be involved in the enzootic circulation of the bacterium as specific biological vectors, whereas greater mouse-eared bats may serve as reservoir hosts since they are capable of developing active bacteremia. On the other hand, *M. myotis* are unlikely to play a significant role in the maintenance of *A. phagocytophilum* and *Rickettsia* spp. Our results highlight the need for further filed studies on the vector potential of spinturnicid mites and their role in the ecology and epidemiology of bacterial infections associated with vespertilionid bats, especially regarding the genus *Bartonella*.
